# Quantifying NO_x_ point sources with Landsat and Sentinel-2 satellite observations of NO_2_ plumes

**DOI:** 10.1073/pnas.2317077121

**Published:** 2024-06-24

**Authors:** Daniel J. Varon, Dylan Jervis, Sudhanshu Pandey, Sebastian L. Gallardo, Nicholas Balasus, Laura Hyesung Yang, Daniel J. Jacob

**Affiliations:** ^a^School of Engineering and Applied Sciences, Harvard University, Cambridge, MA 02138; ^b^GHGSat, Inc., Montréal, QC H2W 1Y5, Canada; ^c^Jet Propulsion Laboratory, California Institute of Technology, Pasadena, CA 91109; ^d^Centro Atomico Bariloche, Bariloche, Argentina

**Keywords:** nitrogen oxides, air pollution, point sources, satellites, remote sensing

## Abstract

We show that the Landsat and Sentinel-2 satellites can detect NO_2_ plumes from large point sources at 10 to 60 m pixel resolution in their blue and ultrablue bands. We use the resulting NO_2_ plume imagery to quantify nitrogen oxides (NO_x_) emission rates for several power plants in Saudi Arabia and the United States, including a 13-y analysis of 132 Landsat plumes from Riyadh power plant 9 from 2009 through 2021. NO_2_ in the plumes initially increases with distance from the source, likely reflecting recovery from ozone titration. The fine pixel resolutions of Landsat and Sentinel-2 enable separation of individual point sources and stacks, including in urban background, and the long records enable examination of multidecadal emission trends. Our inferred NO_x_ emission rates are consistent with previous estimates to within a precision of about 30%. Sources down to ~500 kg h^−1^ can be detected over bright, quasi-homogeneous surfaces. The 2009 to 2021 data for Riyadh power plant 9 show a strong summer peak in emissions, consistent with increased power demand for air conditioning, and a marginal slow decrease following the introduction of Saudi Arabia’s Ambient Air Standard 2012.

## Significance

Atmospheric nitrogen oxides (NO_x_) are air pollutants with important implications for air quality, climate, and the biosphere. Satellites have mapped atmospheric NO_2_ concentrations since the 1990s, but with spatial resolution generally too coarse to resolve individual point sources such as power plants. We show here that the Landsat and Sentinel-2 land-surveying satellites can map large NO_2_ plumes at 10 to 60 m resolution in their visible bands and quantify emissions from individual power plants in favorable observing conditions, despite not having been designed for this purpose. Their high spatial resolution enables separation of individual point sources and stacks, and their long records, with global coverage every few days, enable analysis of multidecadal emission trends.

Nitrogen oxides (NO_x_ ≡ NO + NO_2_) play important roles in air quality, radiative forcing, and nitrogen deposition to the biosphere. Natural sources of NO_x_ include lightning, soils, and wildfires. Anthropogenic emissions are mainly from fossil fuel combustion by vehicles and large stationary point sources such as power plants. Dedicated satellite instruments measuring backscattered sunlight in the ultraviolet-visible (UV/Vis) spectral range have provided global mapping of atmospheric NO_2_ concentrations since the 1990s to quantify NO_x_ emissions and trends, with pixel resolution down to 3.5 × 5.5 km^2^ for the highest-resolution TROPOMI instrument launched in 2017 (1). This is generally too coarse to resolve individual NO_x_ point sources except for large, isolated facilities and/or using extensive temporal averaging (2–7). Improved pixel resolution could enable fine-scale monitoring and attribution of air pollution in dense urban environments and in developing countries where pollution controls and monitoring systems are lacking. Here, we demonstrate the ability of the Landsat and Sentinel-2 satellites to quantify strong NO_x_ point sources at 10 to 60 m pixel resolution with single-pass observations and thus monitor facility-level emissions and trends, including in urban background.

Landsat (8) and Sentinel-2 (9) are global land-surveying satellite missions for monitoring terrestrial resources and land use. Landsat has provided continuous global coverage since the launch of Landsat 1 in 1972, with Landsat 8 (2013 to present) and Landsat 9 (2021 to present) forming the current operational constellation. The Landsat satellites are in near-polar sun-synchronous orbit with a 10:00 to 10:30 equatorial crossing time on the descending node. Each carries an operational land imager (OLI) with 185 km swath, 15 to 30 m pixel resolution, and 9 spectral channels ranging from the visible to shortwave infrared. Together, Landsat 8 and 9 provide global coverage every 8 d. Sentinel-2 comprises a pair of satellites, Sentinel-2A (2015 to present) and Sentinel-2B (2017 to present), in polar sun-synchronous orbit with a ~10:30 equatorial crossing time. Their multispectral instruments (MSI) observe in 13 spectral channels from the visible to shortwave infrared, with 10 to 60 m spatial resolution across a 290 km swath. Together, the Sentinel-2 satellites scan the globe every 5 d.

Here we show how the blue (B) and ultrablue (UB) bands of the Landsat OLI and Sentinel-2 MSI can be used to map NO_2_ plumes at 10 to 60 m pixel resolution and quantify individual NO_x_ point sources. The shortwave infrared bands have previously been used to identify methane plumes (10, 11). We focus on large power plants in scenes with favorable (bright and/or quasi-homogeneous) surface conditions but also demonstrate the ability to resolve point sources over darker surfaces and against an urban backdrop. We present a long-term record of emissions from a large oil- and gas-fired power plant near Riyadh, Saudi Arabia including 132 plumes detected over 13 y from 2009 through 2021.

## Results

**Fig. 1.**
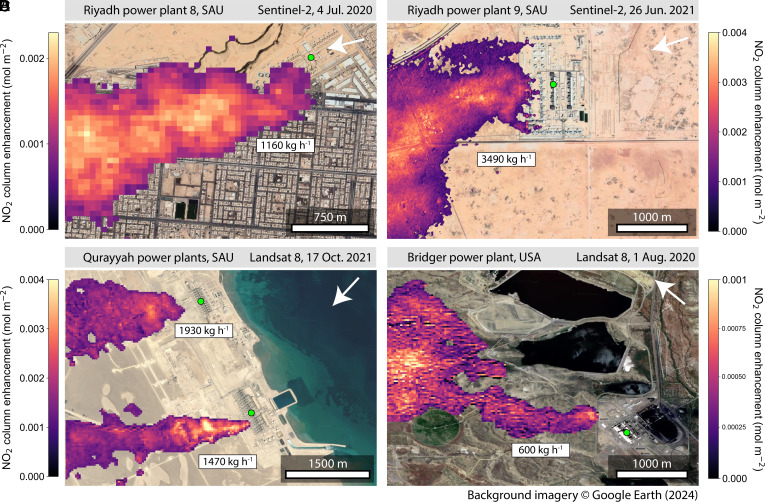
Sample Sentinel-2 and Landsat 8 retrievals of NO_2_ plume column enhancements from five power plants in Saudi Arabia and the United States. (*A*) Riyadh power plant 8, Saudi Arabia (24.597°N, 46.572°E), based on the 60-m Sentinel-2 UB band. (*B*) Riyadh power plant 9, Saudi Arabia (24.950°N, 47.065°E), based on the 10-m Sentinel-2 B band. (*C*) Al-Qurayyah power plants, Saudi Arabia (25.844°N, 50.126°E), and (*D*) Jim Bridger power plant, Wyoming (41.738°N, 108.786°W), based on the 30-m Landsat 8 UB band. The white arrows indicate GEOS-FP 500-m wind direction. The plumes are masked using a percentile threshold and spatial smoothing (*Materials and Methods*). Inferred source rates are indicated *Inset*. The green points mark the centers of the emitting facilities; since NO_x_ is emitted as NO the origin of the detected NO_2_ plume is often a short distance downwind. The color scale varies between panels. Background surface imagery is from © Google Earth.

Fig. 1 shows a selection of NO_2_ plumes from power plants in Saudi Arabia and the United States as observed by Landsat and Sentinel-2. The scenes are generally bright, with surface types ranging in complexity from remote desert (Fig. 1 *B* and *C*) to urban mosaic (Fig. 1*A*) and semiarid steppe (Fig. 1*D*). The retrievals use different instruments and spectral bands to illustrate the range of options available; NO_2_ sensitivity is highest in the ultrablue band, but Sentinel-2’s blue band offers the finest pixel resolution (*Materials and Methods*). The observed plume directions suggest easterly flows consistent with modeled winds from the NASA goddard earth observing system fast processing (GEOS-FP) meteorological product (12). Inventory estimates from the global power emission Database (GPED) (13) and previous TROPOMI-based estimates provide independent evaluation of our estimated source rates. We selected the power plants of Fig. 1 as test cases based on previous reports of strong emissions from GPED and/or remote sensing. Strong NO_x_ emissions from power plants in Riyadh (Fig. 1 *A* and *B*) have been documented by OMI (14) and TROPOMI (2); the Qurayyah power plants (Fig. 1*C*) are together the highest-emitting Saudi Arabian gas plants in the GPED inventory, and Cusworth et al. (15) previously mapped the CO_2_ plume from the Bridger power plant (Fig. 1*D*) on the date we examine here using aerial remote sensing.

Fig. 1*A* shows an NO_2_ plume from Riyadh power plant 8 (24.597°N, 46.572°E) detected by Sentinel-2 on 4 July 2020. Riyadh 8 is a 2.1 GW natural gas and diesel power plant. We estimate a single-pass NO_x_ emission of 1,160 kg h^−1^ for the plant using a cross-sectional flux method (*Materials and Methods*). This is lower than the temporal mean estimates of 2,050 kg h^−1^ from GPED for 2010 and 1,690 ± 710 kg h^−1^ as reported by Beirle et al. (2) from analysis of TROPOMI data for the December 2017 to October 2018 period using the divergence method (*Materials and Methods*). The Sentinel-2 plume is clearly detectable despite the surface variability of the urban scene (roads, buildings), which is removed through the retrieval’s reference scene (*Materials and Methods* and *SI Appendix*, Tables S1 and S2). This demonstrates the potential of high-resolution satellite observations of NO_2_ to map air pollution at the city-block scale.

Fig. 1*B* shows a Sentinel-2 detection of the NO_2_ plume from Riyadh power plant 9 (24.950°N, 47.065°E) on 26 June 2021. Riyadh 9 is a 1.7 GW power plant fueled by crude oil, natural gas, and diesel. It is located about 50 km east of Riyadh. NO_x_ emissions from the plant are large and routinely detectable over its bright and uniform surroundings. The sample observation shown in Fig. 1*B* demonstrates the ability of Sentinel-2 to resolve emissions from individual power plant exhaust stacks using the blue band with 10-m pixel resolution. For this detection, we estimate a total NO_x_ emission of 3,490 kg h^−1^ for the plant. We present below the 13-y history of Riyadh 9 NO_x_ emissions from 2009 to 2021 as observed by Landsat 7 and Landsat 8, including detailed comparison with previous estimates.

Fig. 1*C* shows two large NO_2_ plumes from power plants in the eastern Qurayyah province of Saudi Arabia, detected by Landsat 8 on 17 October 2021. Qurayyah I and II are among the largest gas-fired power plants in the world at 3.9 and 3.8 GW, respectively, and are located less than 3 km apart. Landsat can separate the individual plumes, but TROPOMI could not. We find single-pass emissions of 1,930 kg h^−1^ for Qurayyah I to the north and 1,470 kg h^−1^ for Qurayyah II to the south, similar to the combined GPED estimate of 3,310 kg h^−1^.

Fig. 1*D* shows an NO_2_ plume from the 2.4 GW Jim Bridger coal-fired power plant in Wyoming, detected by Landsat 8 on 1 August 2020. NO_x_ plumes from US power plants are not easily detectable by Landsat and Sentinel-2 due to widespread use of NO_x_ emission controls. The Bridger power plant is outfitted with low-NO_x_ burner technology. Of 11 clear-sky Landsat 8 passes free of condensed water vapor plumes in 2020, we detect only one NO_2_ plume from the power plant, at 11:55 local time on 1 August 2020, under low-wind conditions; the 10-m wind *U*_10_ was 1.6 m s^−1^ based on GEOS-FP. The US EPA clean air markets program database (CAMPD) reports NO_x_ emissions of 800 kg h^−1^ for that hour from the Bridger continuous emission monitoring system (CEMS). We estimate 600 kg h^−1^ from Landsat 8, which may reflect a low bias from ozone titration (discussed below). NO_x_ is mainly emitted as NO, and conversion to NO_2_ in the fresh plume from oxidation by ozone would be delayed if ozone is titrated (16). The plume starts to be detectable a few hundred meters downwind of the exhaust stacks, likely also due to ozone titration, and the initially slender plume eventually widens when it encounters stronger turbulence, possibly due to complex topography. The Bridger case demonstrates that relatively low NO_x_ emissions can be detected in complex scenes if winds are low and if a good reference scene for the retrieval is available (here 17 August 2020; *Materials and Methods*).

Table 1 summarizes the comparisons of our source rate results with previous estimates for the five power plants of Fig. 1. Our estimates show a mean low bias of 11%, which may reflect delayed NO_2_ formation in the fresh plume due to ozone titration. The mean absolute deviation between estimates is about 30%, and we take this to represent a lower limit on the source rate retrieval precision. An error SD of 30% or more is consistent with what one would expect from uncertainty in the wind speed (17) and NO_x_/NO_2_ ratio (4).

**Table 1.** Comparison with previous NO_x_ emission estimates for the power plants of Fig. 1

	This work	Previous work	
	Estimate(kg h^−1^)^*^	Estimate(kg h^−1^)^†^	Source
Riyadh 8	1,160	1,690, 2,050	Beirle et al. (2), GPED
Riyadh 9	3,300 ± 1,080	2,230, 3,900	Beirle et al. (2, 4)
Qurayyah I	1,930	3,310^‡^	GPED
Qurayyah II	1,470		
Bridger	600	800	US EPA (CEMS) CAMPD

^*^Values are for the instantaneous plumes shown in Fig. 1, except for the Riyadh 9 estimate, which is based on the December 2017 to October 2018 period of Fig. 3.

^†^Values reflect different time periods. GPED estimates are for 2010. Estimates by Beirle et al. (2) are for December 2017 to October 2018. Estimates by Beirle et al. (4) are for 2018. The US EPA CEMS estimate is for 11:00 to 12:00 local time on 1 August 2020.

^‡^Combined emission for both power plants.

**Fig. 2.**
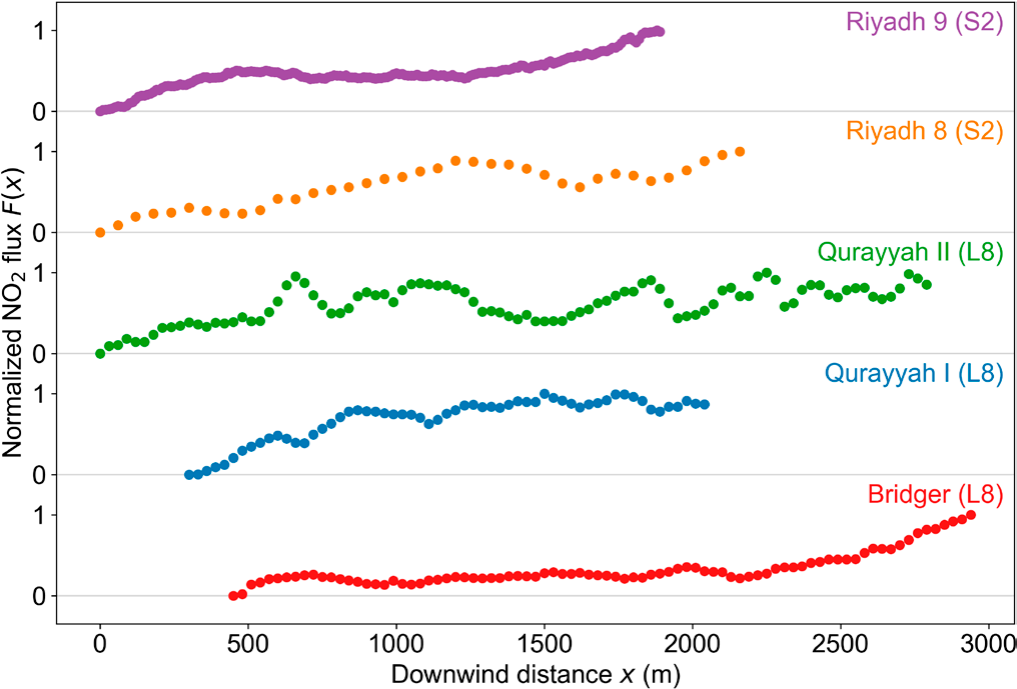
Normalized NO_2_ flux F(x) versus downwind distance *x* from the power plant for the five plumes of Fig. 1. The normalization is a min-max rescaling of F(*x*) to the [0, 1] interval. The solid points represent complete plume cross-sections without truncation at the edge of the retrieval domain.

Fig. 2 shows normalized NO_2_ fluxes as a function of distance downwind of the power plant for the five plumes of Fig. 1. In all cases, the NO_2_ cross-sections tend to grow with distance, which we attribute to gradual recovery from ozone titration as background ozone is entrained into the diluting plume. None of the plumes show an eventual decrease downwind that would reflect NO_x_ oxidation, and that may be explained by the short aging times (~10 min) relative to the lifetime of NO_x_ against oxidation (~1 h). By contrast, much larger and coarsely resolved OMI and TROPOMI plumes show declining NO_2_ with distance from the source due to NO_x_ oxidation (14, 18). The Riyadh 8 and Qurayyah plumes show early recovery from ozone titration followed by steady NO_2_ fluxes, while the Riyadh 9 and Bridger plumes with increasing NO_2_ fluxes suggest delayed recovery and our source rate estimate would then be a lower limit.

**Fig. 3.**
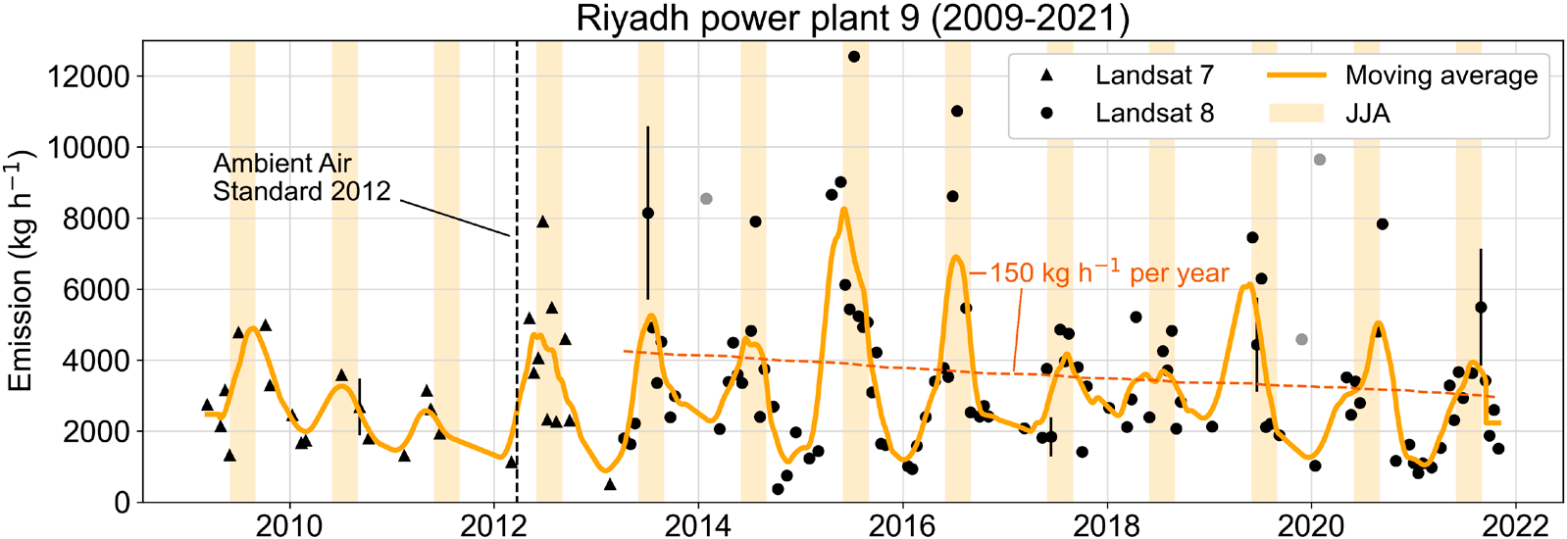
Time series of single-pass NO_x_ emissions from Riyadh power plant 9 inferred from 13 y of Landsat 7 and Landsat 8 observations from 2009 through 2021. NO_2_ plumes are detectable on 28 Landsat 7 passes and 104 Landsat 8 passes. The dashed vertical line indicates the adoption of the Saudi Arabian Ambient Air Standard 2012. The orange line shows the 90-d moving average of a daily interpolation of the single-pass estimates, excluding three outliers in January 2014, January 2020, and November 2019 (marked as gray circles) to better convey the overall seasonal trend. Five sample points are plotted with error bars indicating 1σ uncertainty of 30% (see text). The dashed brown line shows the least-squares trend in emissions from 2013 through 2021. The yellow shaded regions indicate the local summer June–July–August, (JJA) months.

Fig. 3 shows a 13 y history of NO_x_ emissions from Riyadh power plant 9 based on 2009 to 2021 Landsat observations. We quantified a total of 132 plumes for that power plant in cloud-free scenes over this period, including 28 from Landsat 7 (2009 to 2013) and 104 from Landsat 8 (2013 to 2021). We only consider passes for which the GEOS-FP 10-m wind *U*_10_ exceeds 2 m s^−1^ in order to exclude observations with uncertain wind direction when selecting reference scenes for the retrieval (*Materials and Methods*). Landsat 7 shows sparser detections than Landsat 8 because its retrievals are significantly noisier due to an instrument failure in 2003 that led to the loss of about 25% of image pixels (*SI Appendix*, Fig. S2). We therefore consider Landsat 7 data only before Landsat 8 became operational in March 2013. Plumes were clearly detectable in 76% of Landsat 8 passes. Nondetections cannot generally be assumed to reflect low or null emissions because the retrieval precision and corresponding detection limit can vary strongly from pass to pass with the quality of the best available reference scene. The period-average values reported below are therefore based only on detected plumes.

We find that NO_x_ emission from the power plant was 3,480 ± 2,210 kg h^−1^ (mean ± SD) from 2009 to 2021. Our mean NO_x_ emission estimate for the year 2010 is 2,320 ± 680 kg h^−1^, higher than the GPED value of 1,130 kg h^−1^. Beirle et al. (2) reported a mean emission of 2,230 kg h^−1^ for December 2017 to October 2018, and we obtain 3,300 ± 1,080 kg h^−1^ for that period. Beirle et al. (4) made several improvements to their earlier methods, including better estimates of NO_x_/NO_2_ ratios (which we implement here) and NO_x_ chemical lifetime, and reported an updated mean estimate of 3,900 kg h^−1^ for the year 2018, 18% higher than our 3,300 kg h^−1^ estimate. This may be due to the ozone titration bias discussed above and/or to diurnal variability in power generation if emissions are higher during afternoon TROPOMI passes (~13:00 local time) than morning Landsat passes (~10:15 local time) due to increased air conditioning (AC) power demand. Such diurnal emission variability has previously been observed for power plants in the United States (19, 20), China (21), and Qatar (22).

Landsat infers strong seasonal variability in emissions from Riyadh power plant 9, with much higher levels during summer (June-July-August, JJA) than winter (December–January–February, DJF), again likely due to AC power demand. Landsat 8 observes a mean summer-to-winter emission ratio of 1.9, or 3.4 when removing two outlier plumes in January 2014 and January 2020 from the average (Fig. 3). These ratios are higher than the ratio of ~1.6 reported by Lange et al. (23) for total NO_x_ emissions in the Riyadh urban area, as would be expected from weaker seasonality in the transport sector. Saudi Arabia introduced new air quality standards in March 2012 (Ambient Air Standard 2012). Our retrievals show that NO_x_ emissions spiked that summer and then averaged 3,570 kg h^−1^ from 2013 to 2021, with a weak decreasing trend of −150 [−330, 30] kg h^−1^ per year (*p* = 0.1) based on Landsat 8. The 33% increase from the pre-2012 average of 2,670 kg h^−1^ may reflect increased power generation at Riyadh power plant 9 to offset decreased generation elsewhere, and the gradual post-2012 decline may reflect improvement in pollution control technology.

## Discussion

Our work demonstrates the capability to detect and quantify strong NO_x_ point sources with Landsat and Sentinel-2 multispectral satellite observations. We used the instruments’ blue and ultrablue bands to map NO_2_ plumes from individual power plants at 10 to 60 m resolution and infer source rates, focusing on a few large Saudi Arabian and US facilities. The NO_2_ plumes we examined tended to grow downwind, likely reflecting recovery from ozone titration and demonstrating the potential to characterize the chemical dynamics of individual NO_x_ point source plumes from space. The source rates we estimated with Sentinel-2 and Landsat are consistent with previous estimates to within a precision of about 30%, but biased low by about 10% due to ozone titration in the fresh plume. Our 13-y analysis of NO_2_ plumes from Riyadh power plant 9 illustrates how Landsat can enable both seasonal and multidecadal monitoring of emissions and trends for individual NO_x_ point sources.

The limitations of Landsat and Sentinel-2 NO_2_ retrievals must be recognized. The retrievals are most successful over bright, uniform surfaces. Over these surfaces, a source rate detection limit of about 500 kg NO_x_ h^−1^ can be inferred from the consistent detection of emissions from Riyadh power plant 9, for which our lowest source rate estimate was 370 kg h^−1^. Identifying a good reference scene to remove albedo-related artifacts is more challenging for complex surfaces, but we were able to sporadically detect plumes over darker surfaces (Bridger) and an urban mosaic (Riyadh 8). Additionally, the retrievals cannot be applied to scenes containing large aerosol plumes, such as condensed water vapor in cold conditions or carbon particles in poorly performing combustion systems (*Materials and Methods*). The most promising targets for future work may therefore be gas-fired power plants in warm arid regions.

Quality of the reference scenes is presently the main factor limiting our ability to detect NO_2_ plumes with Landsat and Sentinel-2 in more diverse observing conditions. This could be addressed in the future by applying statistical learning to the long data records. Precision in the inferred source rates may be limited by uncertainty in wind speed (17). Low bias from ozone titration could be addressed by tracking the plumes further downwind in adjacent tiles, considering that in all cases of Fig. 1, the plumes exit the tile before they have dissipated to below the detection limit (*Materials and Methods*). An effective tile size of ~20 km would likely be sufficient for this purpose. Mining of the Landsat and Sentinel-2 global records would give a better characterization of detection limits and enable better comparison to the large TROPOMI point source dataset developed by Beirle et al. (4). These records extend back to the launches of Sentinel-2A in 2015 and Landsat 4 in 1982 (when the Landsat blue band was introduced). Sentinel-2 and Landsat NO_2_ column retrievals could be directly validated by comparison with ground-based spectrometers stationed downwind of large NO_x_ point sources. It may be possible to extend the Landsat/Sentinel-2 NO_2_ capability to other multispectral Earth observation satellites with coarse UV/Vis spectral bands, including instruments in low-Earth orbit with up to daily revisits (e.g., Sentinel-3, MODIS) and in geostationary orbit with up to 5-min revisits (e.g., GOES, Himawari-8), though many of these instruments have coarser pixel resolution. Hyperspectral surface mapping instruments such as EMIT (24), PRISMA (25), and EnMAP (26) have ultrablue bands with ~10 nm spectral resolution that should enable better quantification of NO_x_ point sources (27), albeit with lower spatiotemporal coverage than Landsat and Sentinel-2.

## Materials and Methods

### Sentinel-2 and Landsat Data.

**Fig. 4.**
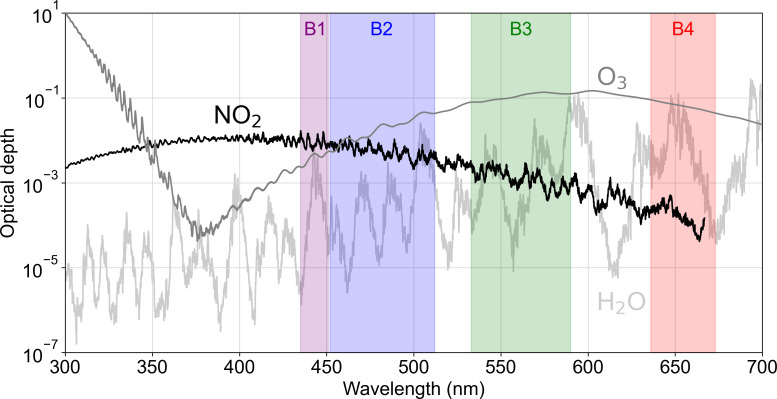
Slant-column optical depths of NO_2_, water vapor (H_2_O), and ozone (O_3_) in the 300 to 700 nm UV/Vis spectral range, based on absorption cross-sections from Vandaele et al. (32) for NO_2_, HITRAN2016 for H_2_O, and Gorshelev et al. (33) and Serdyuchenko et al. (34) for O_3_. The optical depth calculation is performed for a solar zenith angle of 45° with nadir satellite viewing geometry and vertical profiles from the US Standard Atmosphere (35). The shaded areas represent Landsat 8 spectral bands B1 (ultrablue/UB, 435 to 451 nm), B2 (blue/B, 452 to 512 nm), B3 (green/G, 533 to 590), and B4 (red/R, 636 to 673 nm). Sentinel-2 has the same four bands but with slightly different positions and widths. Landsat 7 and earlier do not have the ultrablue band but have nearly identical RGB bands.

Our NO_2_ retrievals use top-of-atmosphere (TOA) reflectances in the Landsat and Sentinel-2 visible bands. We retrieve the reflectance data from Google Earth Engine (GEE) (28) for image tiles of size 6 × 6 km^2^ to 7.5 × 7.5 km^2^, centered on a site of interest. The tile size is limited by the maximum number of pixels that can be retrieved automatically from GEE. The reflectance tiles are delivered along with metadata for acquisition time and viewing/solar zenith angles. Landsat OLI and Sentinel-2 MSI provide four visible bands in the ultrablue (UB, ~430 to 450 nm), blue (B, ~450 to 530 nm), green (G, ~530 to 590 nm), and red (R, ~640 to 680 nm). Landsat 7 and earlier provide only the red, green, and blue (RGB) bands. The Landsat visible bands all have 30-m pixel resolution. The Sentinel-2 RGB bands have 10-m pixel resolution, but the UB band has 60-m resolution. Our retrievals make use of the B and UB bands to quantify NO_2_ column concentrations. The UB band samples stronger NO_2_ absorption lines (Fig. 4) and falls within the spectral range used by TROPOMI and other atmospheric sensors for NO_2_ retrievals (29–31). We use it exclusively for our Landsat 8 retrievals. We use the B band for Landsat 7, which does not have a UB band, and both bands for Sentinel-2 where they have distinct advantages (stronger absorption for UB, but finer pixel resolution for B).

Fig. 4 shows optical depths of NO_2_, water vapor, and ozone in the 300 to 700 nm UV/Vis spectral range, along with Landsat 8 bands 1 to 4. The optical depths are based on absorption cross-sections from Vandaele et al. (32) for NO_2_, the High-resolution TRANsmission molecular absorption (HITRAN2016) database (36) for water vapor, and Gorshelev et al. (33) and Serdyuchenko et al. (34) for ozone, applied to vertical concentration profiles from the US Standard Atmosphere (35). Most of the information on NO_2_ absorption is contained in bands 1 and 2 (UB and B), which have much higher mean optical depths than the other bands (47 times higher in band 1 than in band 4). NO does not have strong absorption features in these bands.

Landsat and Sentinel-2 lack the high spectral resolution needed to retrieve NO_2_ separately from other species in a multiconstituent plume, which could lead to positive (e.g., water vapor absorption) or negative (e.g., condensation scattering) bias in the apparent NO_2_ columns. Coemitted water vapor would be a small source of error because NO_2_ is a much stronger absorber in the ultrablue; the mean optical depth of the US Standard Atmosphere background NO_2_ column (1.4 × 10^−4^ mol NO_2_ m^−2^) in Landsat 8 Band 1 is ~10 times greater than that of the background water vapor column (7.9 × 10^2^ mol H_2_O m^−2^), and >100 times greater with the addition of a 0.002 mol NO_2_ m^−2^ enhancement as observed downwind of several power plants in Fig. 1 (see next section). Coemitted aerosols are another source of uncertainty and may introduce larger errors, as is typical of satellite NO_2_ retrievals (31). We manually inspect each Landsat/Sentinel-2 satellite image to remove from our analysis scenes containing plumes of condensed water or black carbon, which may appear in cold conditions or during power plant startup, and scenes containing clouds.

### NO_2_ Column Retrievals.

Our retrievals infer NO_2_ column enhancements (mol m^−2^) by comparing UB/B reflectances in a scene of interest (“target scene”) with the UB/B reflectances in one or more plume-free reference scenes. The presence of an NO_2_ plume can then be inferred from differences between target and reference. The reference scene should have similar surface features to the target scene but no NO_2_ enhancements; this enables the retrieval to disentangle the spectral signature of the plume from that of surrounding surface features. Here, we adapt the single-band–multi-pass (SBMP) Sentinel-2 methane retrieval introduced by Varon et al. (10) to NO_2_, using the UB or B band on a satellite pass of interest as target scene and the same band on a different pass as reference scene.

For a target scene on pass *i* and reference scene on pass *j*, we compute the normalized reflectance ratio in a band *k* (UB or B) as

[1] $$r_{k}=\frac{c_{k}R_{i,k}}{R_{j,k}}.$$

Here *R* represents the observed top-of-atmosphere reflectance in the band of choice and *c* is a scale factor to remove scene-wide reflectance offsets in that band between passes, which could arise from surface and/or atmospheric conditions (e.g., temporal variations in surface albedo or water vapor absorption) or from changes in the sun-satellite configuration. Following Varon et al. (10), we compute *c* for individual clear-sky scenes by linear regression of all $R_{j}$ values onto all $R_{i}$ values across the scene. We perform the regression after removing water pixels via the normalized difference water index (NDWI; 37). We also remove plume pixels from the scene before performing the regression, since including them would artificially decrease the NO_2_ absorption signal. This involves a two-step retrieval approach in which a preliminary retrieval (first step) provides a plume mask to remove the plume from the regression in the final retrieval (second step).

Identifying a suitable reference scene for persistent NO_x_ point sources (e.g., power plants) requires knowledge of wind direction. We use 10-m wind direction data for individual scenes from the NASA GEOS-FP product at 0.25° × 0.3125° resolution for passes after 20 February 2014 (when the GEOS-FP record begins), and the GEOS Modern-Era Retrospective Analysis for Research and Applications, version 2 (MERRA-2) product at 0.5° × 0.625° resolution for earlier passes. To prevent plume overlap between target and reference scenes, which would attenuate retrieved NO_2_ enhancements, we require wind direction in the two scenes to differ by at least 60°. We use the 10-m wind for this purpose because its model-measurement uncertainty for plume quantification is well documented (38). The uncertainty is particularly high under low wind conditions, hence we only consider scenes with total 10-m wind speed *U*_10_ > 2 m s^−1^ (except for the Bridger scene of Fig. 1). Ehret et al. (11) found that Sentinel-2 methane retrieval artifacts can be reduced by using a linear combination of reference scenes rather than just one, so here we select 1 to 4 references for each target. The number of references is selected manually for each retrieval, and the passes themselves are selected automatically to maximize similarity with the target, which we evaluate from the RMSE between the R band of the target pass and all other passes within a year of it.

We use the Beer–Lambert law to infer NO_2_ slant column enhancements ΔΩ (mol m^−2^) from $r_{k}$ as

[2] $$\Delta \Omega =-\frac{\ln(r_{k})}{\sigma_{k}},$$

where $\sigma_{k}$ (m^2^ mol^−1^) is the band-averaged NO_2_ absorption cross-section in band *k* (blue or ultrablue) based on spectroscopic measurements by Vandaele et al. (32) at 220 K. To account for higher atmospheric temperatures, we apply a temperature correction to the retrieved columns following van Geffen et al. (31). The correction assumes NO_2_ plume enhancements are in the lowest 1,000 m of the atmosphere and is based on the 500 m air temperature inferred from GEOS-FP or MERRA-2.

We convert the retrieved slant columns to vertical columns $\Delta \Omega_{v}$ (mol m^−2^) through a scattering air mass factor (AMF):

[3] $$\Delta \Omega_{v}=\Delta \Omega /\mathrm{AMF}.$$

The AMF describes the light path as a function of the sun-satellite geometry and the scattering by the surface and atmosphere. It is computed as (39):

[4] $$\text{AMF}={\text{AMF}}_{G}\int_{\text{plume}}w\left(z\right)S\left(z\right)dz,$$

where ${AMF}_{G}=\sec\left(\theta_{s}\right)+\sec\left(\theta_{v}\right)$ is the geometric AMF that depends only on the solar ($\theta_{s}$) and viewing ($\theta_{v}$) zenith angles; $w\left(z\right)$ is a wavelength-dependent scattering weight that describes the sensitivity of the TOA reflectance to NO_2_ at altitude *z* ($w\left(z\right)=1$ for a nonscattering atmosphere), and depends only on *z* in the optically thin case; $S\left(z\right)=\Delta n\left(z\right)/\Delta \Omega_{v}$ is a normalized vertical shape profile for the NO_2_ number density enhancement Δn(z); and the integration is over the depth of the NO_2_ plume. We assume $S\left(z\right)$ for the NO_2_ plume to be a step function for the lowest 1 km of the atmosphere so that the AMF can be approximated as

[5] $$AMF\approx {AMF}_{G}w\left(h\right)$$

with h=500m. We take $w\left(h\right)$ from a look-up table of scattering weights for the OMI NO_2_ satellite instrument with a spectral fitting window of 405 to 465 nm, as a function of surface pressure, albedo, $\theta_{s}$, $\theta_{v}$, and the relative azimuth angle (RAA) between sun and satellite (40). We obtain $\theta_{s}$, $\theta_{v}$, and RAA from the satellite metadata. For each Landsat or Sentinel-2 scene, we obtain surface pressure from GEOS-FP or MERRA-2, and albedo (Lambertian-equivalent reflectance from 405 to 465 nm) from the nearest 0.25° × 0.25° grid cell of the OMI level-2 daily gridded retrieval product (41). The use of one albedo value per scene would introduce retrieval errors for plumes over variable surfaces, which might be addressed in the future through high-resolution albedo products. Values of $w\left(h\right)$ range from 0.6 to 1 for our collection of scenes.

We find that the assumption of an optically thin plume for calculating the scattering weights generally holds for large NO_2_ point sources. For example, a vertical column enhancement $\Delta \Omega_{v}=0.002$ mol m^−2^, as observed for several plumes in Fig. 1, would induce <9% extinction (optical depth τ∼0.091) in the UB band of Landsat 8 under typical winter conditions ($\theta_{s}=45^{\circ }$, $\theta_{v}=0^{\circ }$) for the Riyadh power plant 9 scene, and <8% extinction (τ∼0.076) under typical summer conditions ($\theta_{s}=20^{\circ }$, $\theta_{v}=0^{\circ }$), corresponding in both cases to a plume transmittance >90%.

The spectral range of the OMI scattering weight look-up table (405 to 465 nm) contains the Landsat/Sentinel-2 UB bands (~435 to 450 nm) but only partially overlaps the instruments’ B bands (~450 to 520 nm). Using the look-up table in B-band retrievals for Landsat-7 and Sentinel-2 would bias the NO_2_ columns high by underestimating the mean scattering weight and thus underestimating AMF. We account for this by using Landsat-8, with 30-m resolution in both B and UB bands, to characterize the bias between retrievals in the two bands. In a selection of six retrievals from different sites and times of year, we find mean UB:B NO_2_ ratios of 0.6 to 0.75. We take 0.66 as a representative intermediate value, consistent with the expected B:UB ratio of Rayleigh scattering weights between the two bands, and correspondingly scale down our B retrievals to apply the correction.

### Source Rate Retrieval.

Quantification of NO_x_ point sources with satellite observations was previously done by mass balance or fitting a Gaussian plume model to time averages of retrieval scenes from OMI (42, 43) and TROPOMI (44, 45). Beirle et al. (2) introduced a flux-divergence approach to quantify point sources directly from TROPOMI NO_2_ observations and two-dimensional reanalysis winds, and Beirle et al. (3, 4) applied this to construct a global catalog of large NO_x_ point sources. These analyses examined very large plumes extending over tens of km and accounted for loss of NO_x_ by oxidation within the plume.

Here we seek to quantify emissions from single-pass satellite observations of turbulent NO_2_ plumes at ≤60 m pixel resolution and extending over scenes only a few km across, corresponding to a plume aging time of the order of 10 min. This requires a different source rate retrieval because the instantaneous plumes cannot be assumed Gaussian and because the flux divergence approach fails at sub-km scales in the absence of direct wind measurements around the divergence contour (17). Ozone titration in the fresh plume would delay conversion of emitted NO to NO_2_ (16). NO_x_ oxidation is not expected to be significant on the short time scales considered here.

Our approach is to use a cross-sectional flux (CSF) method to quantify the evolution of NO_2_ fluxes in the plume as a function of distance downwind of the source, thus tracking the effects of both ozone titration and NO_x_ oxidation. The CSF method (17, 46) applied to an NO_2_ column retrieval field $\Delta \Omega_{v}$ [mol m^−2^] computes the NO_2_ flux F(x) [kg NO_2_ h^−1^] at a distance *x* downwind of the source as the product of the cross-plume integral column transect *C* [mol m^−1^] and representative wind speed *U* [m s^−1^]:

[6] $$F\left(x\right)=M_{{\text{NO}}_{2}}U\int_{a}^{b}\Delta \Omega_{v}\left(x,y\right)dy=M_{{\text{NO}}_{2}}UC,$$

where *y* is the cross-plume direction perpendicular to the plume axis *x*, $M_{{\mathrm{NO}}_{2}}=0.046$ kg mol^−1^ is the NO_2_ molecular weight, and *a* and *b* are plume boundaries defined from a binary plume mask distinguishing plume from background. We take *U* to be the 500-m wind speed U_500_ from the GEOS-FP or MERRA-2 meteorological fields. We define the orientation of the cross-plume axis *y* by inspection of the plume, from a weighted average of pixel coordinates with the masked NO_2_ enhancements as weights (17). We build the binary plume mask by applying a Gaussian filter with a 1 to 3-pixel kernel to the retrieved columns and then thresholding at the 75th to 90th percentile, similar to the approach of Varon et al. (47) for methane plumes. The masks are manually cropped to remove surface-related retrieval artifacts as necessary.

This approach quantifies emissions from facility-scale point sources as the combined total emission of equipment-scale point sources (e.g., power plant exhaust stacks) within the facility, which may or may not be individually resolvable in the NO_2_ retrieval field. The ability to resolve equipment-scale point sources depends on their spacing relative to the satellite pixel resolution (10 to 60 m), whether ozone titration delays NO_2_ formation directly downwind of the source, and the configuration of equipment relative to the wind direction, since power plant exhaust stacks aligned with the wind direction would produce overlapping plumes.

Results presented in Fig. 2 show that F(x) initially increases with distance downwind of the source, which we attribute to ozone titration (16). The plumes are too short to detect a decrease of F(x) attributable to NO_x_ oxidation. We take the mean of the five highest values of F(x) along the plume axis ($F_{max}$) as our best estimate of the source rate *Q* [kg NO_x_ h^−1^ as NO_2_]:

[7] $$Q=\alpha F_{\max},$$

where *α* [mol mol^−1^] is the NO_x_/NO_2_ concentration ratio in the plume at the distances downwind where $F_{max}$ is calculated. We assume α=1.38 mol mol^−1^ as representative of the plume air after it has recovered from ozone titration (4). The NO_x_ source rate is expressed as equivalent kg NO_2_ to follow standard practice in the emission inventory community.

## Supplementary Material

Appendix 01 (PDF)

## Data Availability

The Landsat and Sentinel-2 top of atmosphere data for this study are available through GEE (https://earthengine.google.com) (28). The GEOS-FP and MERRA-2 wind data are available through the NASA Climate Data Services portal (https://www.nccs.nasa.gov/services/climate-data-services) (12). The HITRAN line spectra are available through the HITRANonline database (https://hitran.org/) (36). All other data are included in the manuscript and/or *SI Appendix*.
