# Supplementary material for: Quantifying NOx point sources with Landsat and Sentinel-2 satellite observations of NO2 plumes
Source: Proc Natl Acad Sci U S A. 2024 Jun 24;121(27):e2317077121. doi: 10.1073/pnas.2317077121 (PMC11228473; doi:10.1073/pnas.2317077121)
Supplement: Supplementary file 1 — Appendix 01 (PDF) [file pnas.2317077121.sapp.pdf]

PNAS

Supporting Information for

Quantifying NO_x point sources with Landsat and Sentinel-2 satellite observations of NO_2 plumes

Daniel J. Varon[1]*, Dylan Jervis[2], Sudhanshu Pandey[3], Sebastian L. Gallardo[4], Nicholas Balasus[1], Laura H. Yang[1], Daniel J. Jacob[1]

[1] School of Engineering and Applied Sciences, Harvard University, Cambridge, United States.
[2] GHGSat, Inc., Montréal, Canada.
[3] Jet Propulsion Laboratory, California Institute of Technology, Pasadena, United States.
[4] Centro Atomico Bariloche, Bariloche, RN, Argentina.

*Corresponding author: Daniel J. Varon

Email: danielvaron@g.harvard.edu

This PDF file includes:

Supporting Figures S1 and S2
Supporting Tables S1 and S2

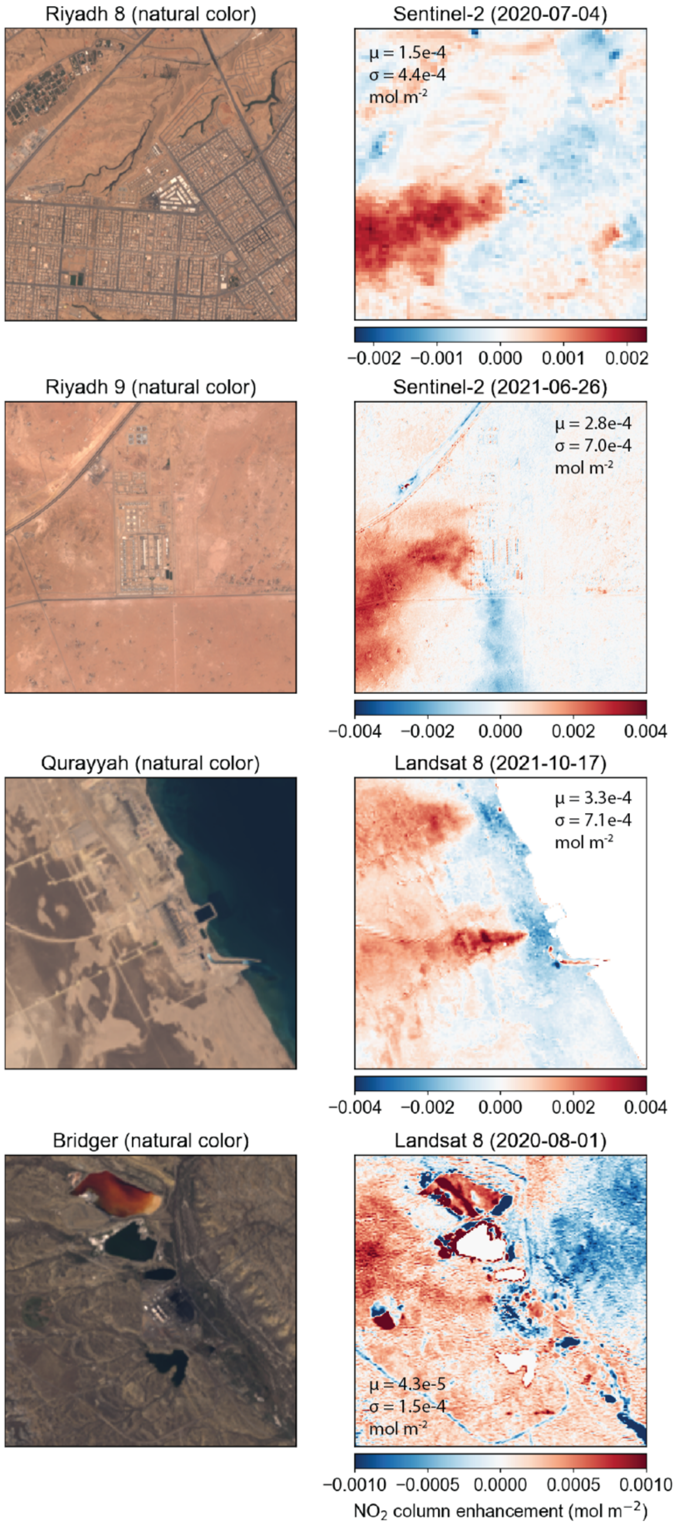

Figure S1. Supporting images for the power plant scenes of Figure 1. (Left column) Satellite natural color composites. (Middle column) Unmasked NO_2 retrieval fields in mol m^{-2}; text insets show the mean (μ) and standard deviation (σ) of the retrieved NO_2 columns after masking out the plume. (Right column) Binary plume masks.

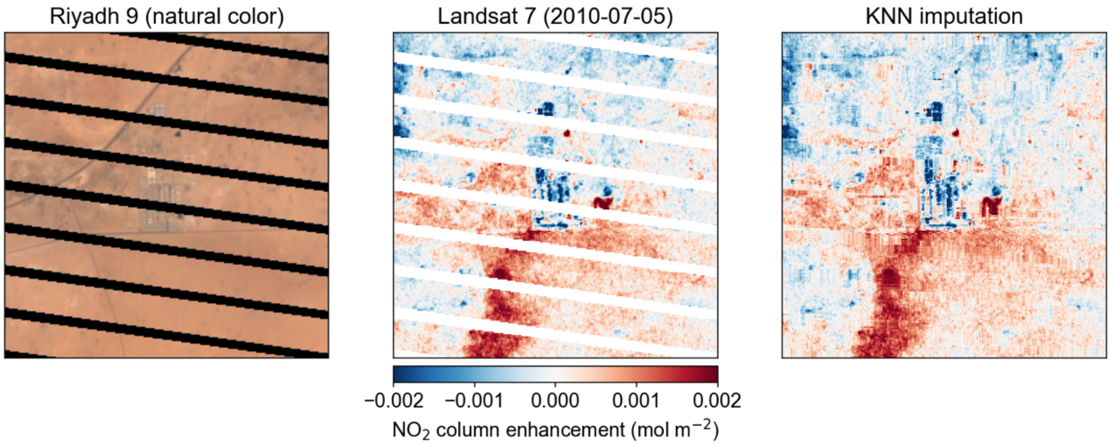

Figure S2. Sample Landsat 7 NO_2 retrieval for Riyadh power plant 9. (Left) Natural color composite with missing pixels in black. (Middle) NO_2 retrieval field with missing pixels in white. (Right) NO_2 retrieval field after gap-filling with a KNN imputer.

Table S1. Supporting information for the retrievals of Figure 1.

Figure	Site	Satellite	Band	Latitude	Longitude	Target Date (yyyy-mm-dd)	Reference Date(s) (yyyy-mm-dd)
1a	Riyadh 8	S2	B1 (UB)	24.597	46.572	2020-07-04	2020-08-03<br>2020-06-04<br>2020-04-25<br>2020-04-10
1b	Riyadh 9	S2	B2 (B)	24.950	47.065	2021-06-26	2021-06-16
1c	Qurayyah	L8	B1 (UB)	25.845	50.126	2021-10-17	2021-11-02<br>2021-10-01<br>2021-12-04
1d	Bridger	L8	B1 (UB)	41.740	-108.790	2020-08-01	2020-08-17

Table S2. Supporting information for the Riyadh power plant 9 retrievals of Figure 3.

Plume Id	Satellite	Band	Target Date (yyyy-mm-dd)	Reference Date (yyyy-mm-dd)	NO_x Emission Rate (kg/h)
0	L8	B1 (UB)	2021-10-31	2021-10-15	1500
1	L8	B1 (UB)	2021-10-15	2021-10-31	2600
2	L8	B1 (UB)	2021-09-29	2021-10-15	1870
3	L8	B1 (UB)	2021-09-13	2021-09-29	3430
4	L8	B1 (UB)	2021-08-28	2021-09-29	5490
5	L8	B1 (UB)	2021-07-27	2021-09-29	3640
6	L8	B1 (UB)	2021-06-25	2021-06-09	2940
7	L8	B1 (UB)	2021-06-09	2021-06-25	3670
8	L8	B1 (UB)	2021-05-24	2021-05-08	2310
9	L8	B1 (UB)	2021-05-08	2021-05-24	3290
10	L8	B1 (UB)	2021-04-06	2021-05-08	1530
11	L8	B1 (UB)	2021-03-05	2021-02-01	980
12	L8	B1 (UB)	2021-02-01	2021-03-05	1100
13	L8	B1 (UB)	2021-01-16	2021-02-17	820
14	L8	B1 (UB)	2020-12-31	2021-01-16	1110
15	L8	B1 (UB)	2020-12-15	2021-01-16	1620
16	L8	B1 (UB)	2020-10-28	2020-10-12	1160
17	L8	B1 (UB)	2020-09-10	2020-08-25	7840
18	L8	B1 (UB)	2020-08-25	2020-09-10	4800
19	L8	B1 (UB)	2020-06-22	2020-05-05	2790
20	L8	B1 (UB)	2020-06-06	2020-05-05	3410
21	L8	B1 (UB)	2020-05-21	2020-05-05	2460
22	L8	B1 (UB)	2020-05-05	2020-05-21	3520
23	L8	B1 (UB)	2020-01-30	2019-12-29	9650
24	L8	B1 (UB)	2020-01-14	2019-12-29	1020
25	L8	B1 (UB)	2019-11-27	2019-12-13	4580

26	L8	B1 (UB)	2019-09-08	2019-10-26	1890
27	L8	B1 (UB)	2019-08-07	2019-07-22	2210
28	L8	B1 (UB)	2019-07-22	2019-08-07	2120
29	L8	B1 (UB)	2019-07-06	2018-08-04	6290
30	L8	B1 (UB)	2019-06-20	2019-07-22	4430
31	L8	B1 (UB)	2019-06-04	2019-05-03	7460
32	L8	B1 (UB)	2019-01-11	2018-12-10	2120
33	L8	B1 (UB)	2018-09-21	2018-09-05	2830
34	L8	B1 (UB)	2018-09-05	2018-09-21	2070
35	L8	B1 (UB)	2018-08-20	2018-06-17	4830
36	L8	B1 (UB)	2018-08-04	2018-07-03	3720
37	L8	B1 (UB)	2018-07-19	2018-06-17	4250
38	L8	B1 (UB)	2018-06-01	2018-06-17	2400
39	L8	B1 (UB)	2018-04-14	2017-07-16	5220
40	L8	B1 (UB)	2018-03-29	2017-10-20	2900
41	L8	B1 (UB)	2018-03-13	2017-10-04	2120
42	L8	B1 (UB)	2018-01-08	2017-12-23	2650
43	L8	B1 (UB)	2017-10-20	2017-10-04	3260
44	L8	B1 (UB)	2017-10-04	2017-10-20	1420
45	L8	B1 (UB)	2017-09-18	2017-06-14	3810
46	L8	B1 (UB)	2017-08-17	2017-06-14	4740
47	L8	B1 (UB)	2017-08-01	2017-06-14	3970
48	L8	B1 (UB)	2017-07-16	2017-05-29	4860
49	L8	B1 (UB)	2017-06-14	2017-05-29	1840
50	L8	B1 (UB)	2017-05-29	2017-06-14	3760
51	L8	B1 (UB)	2017-05-13	2017-04-11	1810
52	L8	B1 (UB)	2017-03-10	2017-01-05	2080
53	L8	B1 (UB)	2016-11-02	2016-10-17	2410
54	L8	B1 (UB)	2016-10-17	2016-11-02	2710

55	L8	B1 (UB)	2016-10-01	2016-08-30	2420
56	L8	B1 (UB)	2016-08-30	2016-10-01	2530
57	L8	B1 (UB)	2016-08-14	2016-04-24	5470
58	L8	B1 (UB)	2016-07-13	2016-08-30	11010
59	L8	B1 (UB)	2016-06-27	2016-06-11	8620
60	L8	B1 (UB)	2016-06-11	2016-06-27	3530
61	L8	B1 (UB)	2016-05-26	2016-06-11	3780
62	L8	B1 (UB)	2016-04-24	2016-06-27	3410
63	L8	B1 (UB)	2016-03-23	2016-04-08	2410
64	L8	B1 (UB)	2016-02-20	2017-01-05	1580
65	L8	B1 (UB)	2016-02-04	2016-01-19	930
66	L8	B1 (UB)	2016-01-19	2016-02-04	1020
67	L8	B1 (UB)	2015-10-31	2015-03-05	1620
68	L8	B1 (UB)	2015-10-15	2015-09-29	1650
69	L8	B1 (UB)	2015-09-29	2015-10-15	4220
70	L8	B1 (UB)	2015-09-13	2015-08-12	3100
71	L8	B1 (UB)	2015-08-28	2015-04-22	5070
72	L8	B1 (UB)	2015-08-12	2015-06-25	4940
73	L8	B1 (UB)	2015-07-27	2015-09-29	5240
74	L8	B1 (UB)	2015-07-11	2015-06-25	12550
75	L8	B1 (UB)	2015-06-25	2015-08-12	5430
76	L8	B1 (UB)	2015-06-09	2015-06-25	6130
77	L8	B1 (UB)	2015-05-24	2015-06-25	9010
78	L8	B1 (UB)	2015-04-22	2015-08-28	8670
79	L8	B1 (UB)	2015-03-05	2014-10-28	1440
80	L8	B1 (UB)	2015-02-01	2014-12-15	1240
81	L8	B1 (UB)	2014-12-15	2015-02-01	1970
82	L8	B1 (UB)	2014-11-13	2014-10-28	760
83	L8	B1 (UB)	2014-10-12	2014-10-28	370

84	L8	B1 (UB)	2014-09-26	2014-07-24	2690
85	L8	B1 (UB)	2014-08-25	2014-06-06	3740
86	L8	B1 (UB)	2014-08-09	2014-07-08	2410
87	L8	B1 (UB)	2014-07-24	2014-07-08	7900
88	L8	B1 (UB)	2014-07-08	2014-07-24	4820
89	L8	B1 (UB)	2014-06-06	2014-07-08	3360
90	L8	B1 (UB)	2014-05-21	2014-04-19	3600
91	L8	B1 (UB)	2014-05-05	2014-05-21	4490
92	L8	B1 (UB)	2014-04-19	2014-05-21	3390
93	L8	B1 (UB)	2014-03-18	2015-02-17	2060
94	L8	B1 (UB)	2014-01-29	2014-02-14	8540
95	L8	B1 (UB)	2013-10-09	2013-08-22	2990
96	L8	B1 (UB)	2013-09-23	2013-08-22	2390
97	L8	B1 (UB)	2013-08-22	2013-09-23	4510
98	L8	B1 (UB)	2013-08-06	2013-08-22	3360
99	L8	B1 (UB)	2013-07-21	2013-06-19	4920
100	L8	B1 (UB)	2013-07-05	2013-04-09	8140
101	L8	B1 (UB)	2013-05-18	2013-06-19	2220
102	L8	B1 (UB)	2013-05-02	2014-03-18	1640
103	L8	B1 (UB)	2013-04-09	2013-07-05	1800
104	L7	B1 (B)	2013-02-19	2013-02-03	510
105	L7	B1 (B)	2012-09-28	2013-02-19	2310
106	L7	B1 (B)	2012-09-12	2012-03-04	4600
107	L7	B1 (B)	2012-08-11	2012-07-26	2270
108	L7	B1 (B)	2012-07-26	2011-11-13	5490
109	L7	B1 (B)	2012-07-10	2012-02-01	2330
110	L7	B1 (B)	2012-06-24	2012-06-08	7910
111	L7	B1 (B)	2012-06-08	2012-06-24	4060
112	L7	B1 (B)	2012-05-23	2012-10-14	3640

113	L7	B1 (B)	2012-05-07	2012-03-20	5180
114	L7	B1 (B)	2012-03-04	2012-09-12	1130
115	L7	B1 (B)	2011-06-22	2010-10-09	1930
116	L7	B1 (B)	2011-05-21	2010-10-09	2630
117	L7	B1 (B)	2011-05-05	2011-01-13	3140
118	L7	B1 (B)	2011-02-14	2010-07-05	1320
119	L7	B1 (B)	2010-10-09	2010-01-10	1790
120	L7	B1 (B)	2010-09-07	2009-12-09	2680
121	L7	B1 (B)	2010-07-05	2009-10-22	3580
122	L7	B1 (B)	2010-02-27	2009-09-20	1740
123	L7	B1 (B)	2010-02-11	2009-09-20	1670
124	L7	B1 (B)	2010-01-10	2010-10-09	2460
125	L7	B1 (B)	2009-10-22	2010-07-05	3280
126	L7	B1 (B)	2009-10-06	2010-01-10	4980
127	L7	B1 (B)	2009-07-02	2009-02-08	4780
128	L7	B1 (B)	2009-05-31	2009-05-15	1330
129	L7	B1 (B)	2009-05-15	2009-05-31	3150
130	L7	B1 (B)	2009-04-29	2009-05-15	2140
131	L7	B1 (B)	2009-03-12	2009-04-13	2750
